# Report of the Rochester Hahnemannian Society

**Published:** 1889-03

**Authors:** 


### REPORT OF THE ROCHESTER HAHNEMANNIAN SOCIETY.

The regular annual meeting of the Rochester Hahnemannian Society was held at the office of Dr. W. G. Brownell, January 15th; President R. C. Grant, M. D., in the chair.

Members present: Drs. Grant, Schmitt, Carr, Hoard, Hermance, Baker, Brownell, Johnson. W. S. Lanning, M. D., of this city, was present as visitor.

Officers for 1889 were elected as follows: President, Allen B. Carr, M. D.; Vice-President, Julius G. Schmitt, M. D.;

Secretary and Treasurer, W. H. Baker, M. D.; Censors, J. A. Biegler, M. D., Chairman; R. C. Grant, M. D., and A. C. Hermance, M. D.

Dr. V. A. Hoard was elected delegate to the next meeting of the I. H. A.

Before adjournment, R. C. Grant, M. D., the retiring President, made the following remarks:

"GENTLEMEN OF THE ROCHESTER HAHNEMANNIAN SOCIETY: I cannot resign the office you so kindly intrusted to me a year ago, to my successor, without thanking you for the kindness and courtesy with which I have been ever treated during my term of office. My shortcomings, which I fear have been many, have ever been passed lightly by; and my weakness ever supported by the strength of others, and in return it has been my aim to discharge my duties at least impartially, promptly, and regularly, if not with grace and wisdom. I thank you; I can say no more, I can say no less.

"I feel that this has been a red-letter year in the existence of this Society. We have gained in numbers, we have gained in earnestness and steadfastness of purpose, we have, I trust, gained in wisdom. The first subject of large importance that we had to meet the past year was one which I had the honor to bring before you, and which found ready response in the hearts of all; I refer to the establishment of a Hahnemannian hospital. I am asked almost daily, 'is there need of another hospital in Rochester? we have already two in operation on quite a large basis, and another chartered.' I answer always and promptly, yes; could a city be so full of allopathic and eclectic physicians that there would be no need of a homœopath? The two existing hospitals are allopathic; the newly-chartered institution—if it ever reaches a consummation—will be an eclectic hospital, with a homœopathic name only. We do need a hospital here where we can assure our patients and friends that they can have homœopathic treatment, and where we can demonstrate, as they are demonstrating daily in the noble Woman's Homœopathic Hospital in Philadelphia, the undoubted superiority of absolute Homœopathy. With a hospital under Hahnemannian Homœopathy, if we succeeded in reducing the mortality, in curtailing the course of disease, or in alleviating pain without injuring the patient, or retarding recovery, we would be at once furnished with a weapon that the armor of allopathy or eclecticism could not withstand. Yes, gentlemen, we do need a Hahnemannian hospital in Rochester, and, unless all signs fail, our hopes will soon grow into full fruition.

"The next question was our withdrawal from the Monroe County Homœopathic Society. The only side I wish to view of this is, whether it was more calculated to help or harm our school (that there was any question of a personal nature in the move is not to be thought of).

"We hear the cry that this internal warfare in our school impedes our progress; that, notwithstanding our prodigious growth, we are still a weak minority and that we should forget differences, join hands and go forth together to meet the common foe. Thank Heaven, this cry comes chiefly from those who cry also good Lord, good Devil in their practice. Of course, we are glad to see our numbers increase; we would be glad to see every physician in the land in the active, honest practice of pure Homœopathy. But do we want men enrolled under our banner who teach their patients the use of the hypodermic syringe and directly lead them into a confirmed Opium habit? Do we want men who will assume our name and then say publicly and repeatedly that when they want to cure acute painful diseases they have to resort to physiological doses of empirical prescriptions? Do we want those with us who will sneer at the efficacy of the dynamized drug?

"Whom did the little handful of patriots who so earnestly desired the independence of these United States most dread (and I may add despise) the regular British army, with their open manly resistance and avowed enmity, or the sneaking Tory who professed friendship, that he might lead his unsuspecting brother into the hands of his enemies? And I might also add for whom did the British have the most respect?

"These Tory friends of ours say they are just as good homœopaths as we, that it is merely a difference in the interpretation of the laws. I say no; there can be no honest difference in the interpretation; it is simply and solely acceptance or rejection of the law. They say, too, that it is simply the potency question that divides us; that we are high and they are low. If they were honest, they would blush at this deception themselves; it is begging the question. We all know that the greatest latitude is given as to potency, if potency be used, but there is the rub; a crude drug is not and never was a potency; and an empirical prescription is not and never was an interpretation of the homœopathic law, under whatever circumstances it might be given. One Judas did more to injure our Lord than all His open enemies combined, and so I say, gentlemen, that the support or condoning of weak-kneed homœopaths is an impediment to our growth.

"Every homœopathist who makes an empirical prescription is putting off the millennium. On these grounds I think we were right in the action taken. I believe that there has been in the past five years a more rapid growth in pure Homœopathy than in any previous ten. Not only does the physician, but the layman also, take an interest in the question of what is and what is not pure Homœopathy, and when it is once honestly and earnestly studied and understood, the Homœopathy of Hahnemann is sure to be accepted.

"Even students where a very doubtful kind of Homœopathy is taught are bringing up the question for open discussion in their Societies, and Homœopathy has advocates among the undergraduates who should put many a practitioner to the blush. There is no doubt in my mind that the next decade will see our ranks pretty well thinned of the Tory element, the Judas element, and a vast army of loyal homœopaths upholding the banner of Hahnemann."

